# Factors associated with EMS on-scene time and its regional difference in road traffic injuries: a population-based observational study

**DOI:** 10.1186/s12873-022-00718-1

**Published:** 2022-09-15

**Authors:** Shingo Ito, Hideki Asai, Yasuyuki Kawai, Shunji Suto, Sachiko Ohta, Hidetada Fukushima

**Affiliations:** 1 Department of Emergency and Critical Care Medicine, Nara Medical University, Shijo-cho, 840, Kashihara City, Nara 634-8522 Japan; 2 Department of Emergency, Shiroyama Hospital, Osaka, Japan; 3 Department of Community Medicine, Nara Medical University, Nara, Japan; 4 Research and Development, Health Informatics and Management Professionals, Tokyo, Japan; 5 Department of Pharmaceutical and Medical Business Sciences, Nihon Pharmaceutical University, Tokyo, Japan

**Keywords:** Emergency medical service, Road traffic injury, Emergency response, on-scene time

## Abstract

**Background:**

The outcome of road traffic injury (RTI) is determined by duration of prehospital time, patient’s demographics, and the type of injury and its mechanism. During the emergency medical service (EMS) prehospital time interval, on-scene time should be minimized for early treatment. This study aimed to examine the factors influencing on-scene EMS time among RTI patients.

**Methods:**

We evaluated 19,141 cases of traffic trauma recorded between April 2014 and March 2020 in the EMS database of the Nara Wide Area Fire Department and the prehospital database of the emergency Medical Alliance for Total Coordination of Healthcare (e-MATCH). To examine the association of the number of EMS phone calls until hospital acceptance, age ≥65 years, high-risk injury, vital signs, holiday, and nighttime (0:00–8:00) with on-scene time, a generalized linear mixed model with random effects for four study regions was conducted.

**Results:**

EMS phone calls were the biggest factor, accounting for 5.69 minutes per call, and high-risk injury accounted for an additional 2.78 minutes. Holiday, nighttime, and age ≥65 years were also associated with increased on-scene time, but there were no significant vital sign variables for on-scene time, except for the level of consciousness. Regional differences were also noted based on random effects, with a maximum difference of 2 minutes among regions.

**Conclusions:**

The number of EMS phone calls until hospital acceptance was the most significant influencing factor in reducing on-scene time, and high-risk injury accounted for up to an additional 2.78 minutes. Considering these factors, including regional differences, can help improve the regional EMS policies and outcomes of RTI patients.

**Supplementary Information:**

The online version contains supplementary material available at 10.1186/s12873-022-00718-1.

## Background

The outcome of critical cases that require immediate treatment, such as acute coronary syndrome, stroke, cardiac arrest, and trauma, is time dependent. Particularly, on-scene time is critical for these conditions, with a delay in definitive treatment leading to poor outcomes [1–5]. For trauma victims, studies have shown that time by minute for treatment is associated with the outcome [6–10]. Several factors account for on-scene time among trauma patients [11–13]; however, the actual impact of these factors has not been sufficiently examined. Among traumatic injuries, road traffic injuries (RTIs) require immediate transport and treatment. Although the total number of RTIs has been decreasing due to recent advances in safety technologies, fatalities remain high [14], especially deaths among the elderly [15]. Notably, it is impossible to shorten the distance between the accident site and the hospital, and no matter how quickly emergency medical service (EMS) reacts, there is a limit to the amount of time that can be shortened. Minimizing the on-scene time can therefore aid in improving the outcomes of RTI in prehospital care settings. However, the factors that account for the EMS on-scene time among RTIs are not well investigated because a variety of factors are intricately associated, as EMS performs multiple tasks simultaneously, such as coordinating with other agencies, as well as evaluating and treating patients in the field.

Therefore, this study aimed to quantitively examine the factors influencing EMS on-scene time in RTIs to improve EMS policies and implementation.

## Methods

### Study design and setting

This was a retrospective analysis of a population-based EMS prehospital dataset. The Nara Wide Area Fire Department covers an area of 3,361 km^2^ with a population of 853,307. The area comprises four medical administrative regions: Seiwa, Chuwa, Tohwa, and Nanwa. Seiwa and Chuwa are both urban regions, with land areas of 168.5 km^2^ and 240.8 km^2^ and populations of 338,775 and 367,425, respectively. Meanwhile, Tohwa and Nanwa are both rural areas, with land areas of 657.7 km^2^ and 2,346.9 km^2^ and populations of 198,650 and 64,993, respectively. Each region has a branch office of the Nara Wide Area Fire Department.

### Japanese EMS system

The Japanese EMS system is a part of the fire department, and the universal 119 call dispatches an ambulance with a three-member ambulance crew. The ambulance crew consists of firefighters, equivalent to the emergency medical team (EMT)-B category in the US, and a nationally certified emergency life-saving technician (ELT), equivalent to the EMT-A category. These ELTs can perform life-saving procedures according to local protocols under the direction of medical doctors for cardiac arrest, critical shock, or unconsciousness due to hypoglycemia. They can secure the airway, establish venous access, administer epinephrine, and measure blood glucose level following glucose IV administration.

### Patients and data source

All RTI patients in the study areas registered in the database between April 1, 2014, and March 31, 2020, were evaluated. Among them, those in whom physicians were at the emergency site were excluded because hospital selection or medical procedures by physicians at the site might have affected the on-scene time. The other exclusion criteria were as follows: missing data of high-risk injury, on-scene time, and the number of EMS phone calls until hospital acceptance. We excluded data with vital signs of systolic blood pressure over 261 mmHg, heart rate over 185 beats per minute, and a respiratory rate over 45 breaths per minute, as these were regarded as errors based on our clinical impressions. On-scene time of less than 6 minutes was also treated as an error.

All emergency trauma reports of the Nara Wide Area Fire Department and the EMS database of the emergency Medical Alliance for Total Coordination of Healthcare (e-MATCH) were analyzed. The e-MATCH, which runs on the portable tablet set in the ambulance, is an online system that supports EMS decision transport of a patient to the nearest hospital. Given that the e-MATCH dataset does not contain the details of emergency calls, we merged the e-MATCH dataset and all the emergency trauma reports by dispatch time, age, and sex.

### Variables

The study dataset contained data of the accident location according to the medical administrative regions, patient age and sex, EMS time intervals (response time, on-scene time, and transport time), number of phone calls from EMS until acceptance at a hospital emergency department, the patient’s first vital signs after EMS contact [systolic and diastolic pressure (mmHg), heart rate (beats per minute), and respiratory rate (breaths per minute)]. We also categorized vital signs according to the National Overall Acuity Scale criteria (Additional file 1) [16]: systolic blood pressure less than 90 mmHg, heart rate <50 or ≥ 120 beats per minute, and respiratory rate <10 or ≥ 30 breaths per minute. Elderly was defined as age ≥65 years.

Level of conscious was graded using the Japan Coma Scale (JCS) [17, 18]. JCS is a one-axis coma scale that is used by EMS in Japan as a standard method to evaluate the level of consciousness among emergency patients. JCS has three categories: no eye opening to any stimuli (level 3), eye opening to verbal or pain stimuli (level 2), and spontaneous eye opening (level 1) consistent with grade 4 for eye response of the Glasgow Coma Scale. The JCS is a simple tool applicable for the prediction of neurological outcomes in stroke and trauma patients [17, 18]. In Nara, the EMS adopted the criteria of eye opening with pain stimuli (JCS 30: subcategory of level 2) and no response (level 3) as that for possible severe brain injury. High-risk injury is the mechanism that can cause serious injuries, as defined by the Japanese Fire and Disaster Management Agency (Additional file 1) [16]. Two study investigators independently determined high-risk injury based on text data in the emergency reports. The inter-rater reliability determined with the Cohen’s kappa efficient was 0.71. Disagreements between the two study investigators were resolved by a discussion between them.

### Statistical analysis

The primary outcome measure of this study was EMS on-scene time. Continuous variables are expressed as the median and interquartile range and were compared between groups using the Mann–Whitney U test. Meanwhile, categorical variables are presented as number (%) and were compared using the chi-square test. Comparisons among three or more groups were performed using the Kruskal–Wallis test with Bonferroni adjustment. Generalized linear mixed model (GLMM) analysis controlling for age, sex, high-risk injury, number of EMS phone calls until hospital acceptance, and vital signs at the scene was performed to identify the influencing factors of EMS on-scene time. A random-effect model analysis was additionally performed according to the four administrative medical regions. Data with missing values were analyzed using the pairwise deletion method. All statistical analyses were performed using SPSS ver. 25.0 (SPSS Inc., Chicago, IL, USA). P values of less than 0.05 were considered significant.

## Results

### Population characteristics

**Fig. 1:**
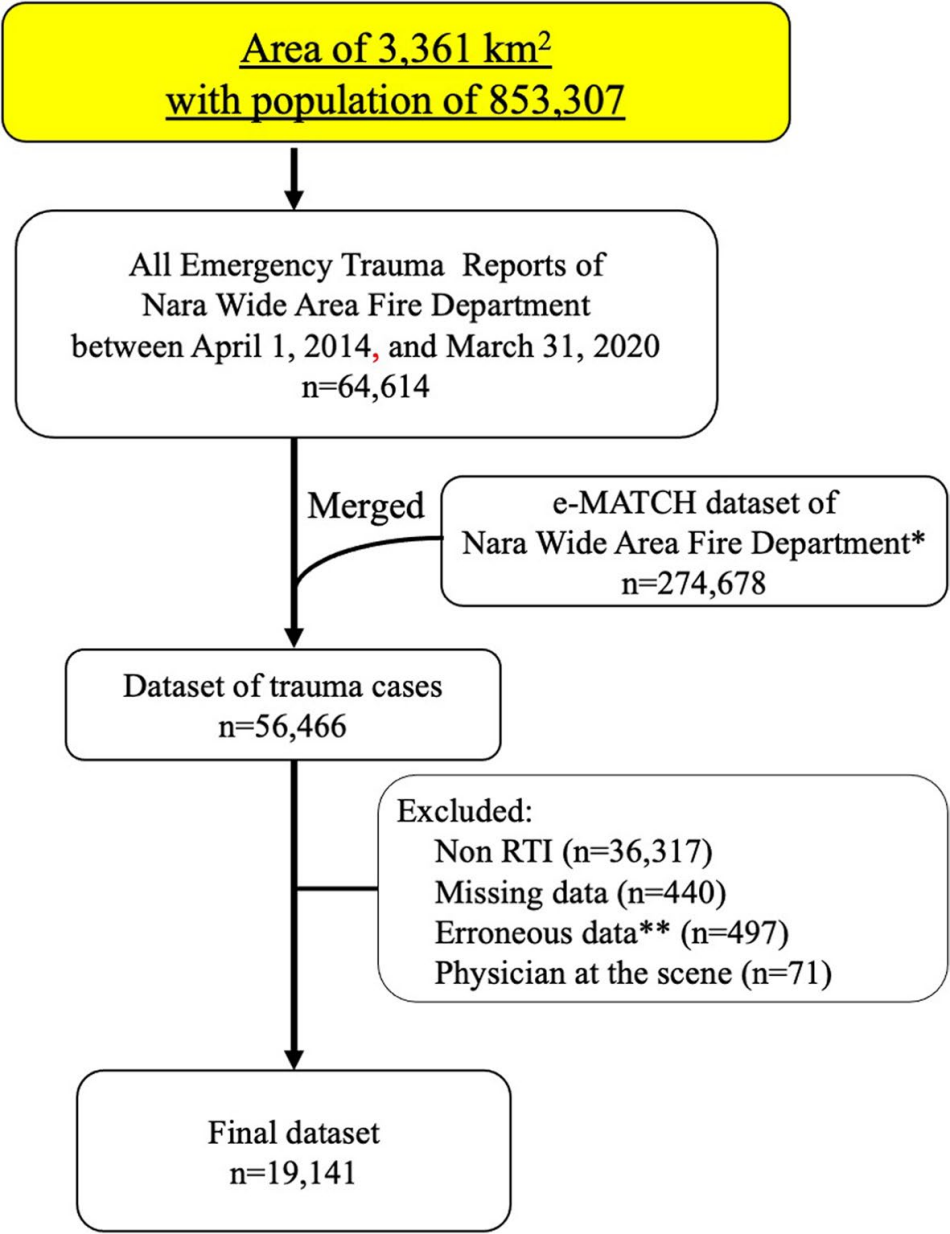
Patient inclusion flowchart. *This dataset includes all emergency cases including those with acute illness. **erroneous data: systolic blood pressure ≥261 mmHg, heart rate ≥185 beats per minute, respiratory rate ≥45 breaths per minute, and on-scene time of less than 6 minutes. e-MATCH, emergency Medical Alliance for Total Coordination of Healthcare; RTI, road traffic injury

The analyzed dataset comprised 56,466 patients. After screening, 19,141 patients were included in the final analysis (Fig. 1). The patient characteristics are shown in Table 1. The majority of the patients were male (55.9%), and the median patient age was 47 years. In total, 5369 (28.0%) patients were elderly. High-risk injury accounted for 5.7% (1091 patients) of RTIs, and 2.4% (454) of patients were transported to tertiary care centers. The median response time, on-scene time, and transportation time were 8, 20, and 10 minutes, respectively. Majority of the RTIs (9281 cases, 48.5%) occurred at daytime during weekdays. The median systolic and diastolic blood pressure were 136 mmHg and 76 mmHg, respectively, and the median heart rate and respiratory rate were 80 beats per minute and 18 breaths per minute, respectively. Less than 20% (14.3% for systolic blood pressure, 16.8% for heart rate, and 16.4% for respiratory rate) of the patients met the criteria for the National Overall Acuity Scale.

**Table 1:** Patient characteristics

	Total		
	*n*=19141	(%)	Missing data
Male	10709	55.9	
Age, years	47 (24–67)		
Elderly (age ≥65 years)	5369	28.0	
High-risk injury, n (%)	1091	5.7	
Transport to tertiary care hospitals	454	2.4	
Number of EMS phone calls until hospital acceptance	1 (1–2)		
Response time, min	8 (7–11)		
On-scene time, min	20 (15–27)		
Transport time, min	10 (6–16)		5
Holiday	6331	33.1	
Daytime (08:00–16:00)	9281	48.5	
Evening (17:00–23:00)	7471	39.0	
Nighttime (00:00–07:00)	2389	12.5	
Conscious level of eye opening by pain stimuli or no response	147	0.8	30
SBP, mmHg	136 (167–158)		166
DBP, mmHg	76 (55–90)		170
HR, beats/min	80 (65–93)		121
RR breaths/min	18 (18–20)		135
SBP less than 90 mmHg	2738	14.3	166
HR <50 or ≥120 beats/min	3216	16.8	121
RR <10 or ≥ 30 breaths/min	3134	16.4	135

*SBP* systolic blood pressure, *DBP* diastolic blood pressure, *HR* heart rate, *RR* respiratory rate, *EMS* emergency medical service

### On-scene time, EMS phone calls until hospital acceptance, and regional differences

**Fig. 2:**
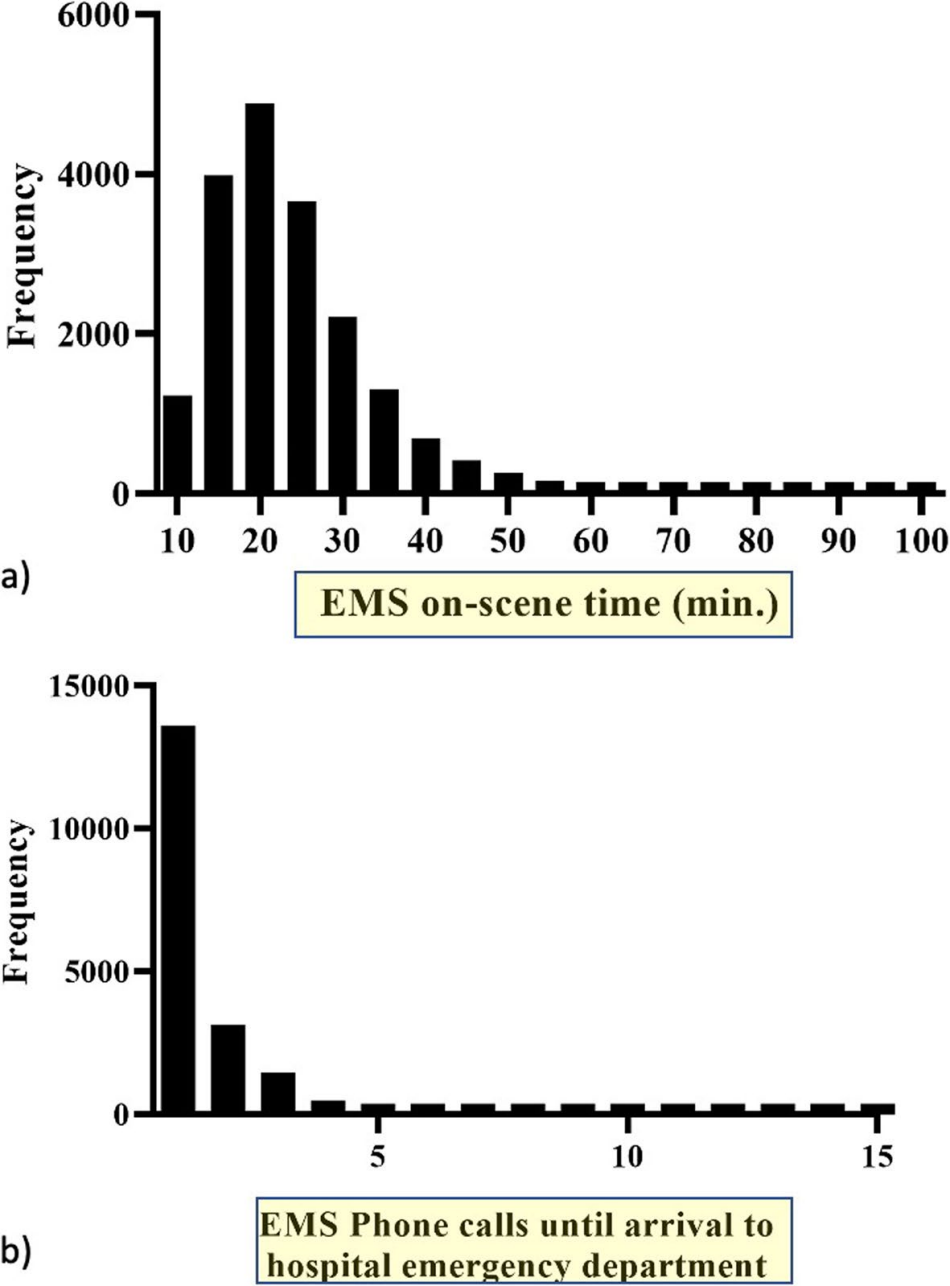
Histogram for (**a**) on-scene time (min.) and (**b**) numbers of EMS phone calls until hospital acceptance. Twenty-six cases with over 105 minutes of EMS on-scene time and 10 cases with over 16 EMS phone calls are not shown in figure a) and b), respectively, for visibility.

Figure 2a shows the histogram of the on-scene time. In most cases (71.9%), on-scene time was within 25 minutes. Figure 2b shows the frequency of EMS calls for hospital acceptance. Hospitals accepted most of the cases with one call. However, 29% of the cases required at least two. Even for high-risk injury, 294 cases required two or more calls until hospital acceptance (Table 2).

**Table 2:** Patient characteristics differentiated by the number of calls until hospital acceptance

	One call		2 or more calls		*p* values
	*n*=13587		*n*=5554		
Age, years	47	(25–67)	46	(24–66)	*P*=0.002
Elderly (≥65 years)	3888	(28.6)	1481	(26.7)	*P*=0.007
Male sex, n (%)	7526	(55.4)	3183	(57.3)	*P*=0.015
High-risk injury, n (%)	797	(5.9)	294	(5.3)	*P*=0.122
Response time, min	8	(7–11)	8	(7–11)	*P*=0.487
On-scene time, min	18	(14–23)	27	(21–35)	*P*<0.001
Transport time, min	9	(5–14)	14	(9–21)	*P*<0.001
Consciousness level of eye opening by pain stimuli or no response	117	(0.9)	30	(0.5)	*P*=0.022
SBP less than 90 mmHg	1820	(13.4)	918	(16.5)	*P*<0.001
HR <50 or ≥ 120 beats/min	2149	(15.8)	1067	(19.2)	*P*<0.001
RR <10 or ≥ 30 breaths/min	2078	(15.3)	1056	(19.0)	*P*<0.001

*SBP* systolic blood pressure, *DBP* diastolic blood pressure, *HR* heart rate, *RR* respiratory rate

Table 3 shows the difference in patient characteristics among the four study regions. The patients in Nanwa area were older than those in the other three areas, and most of the patients in this area were female. The transportation time was also longer in this area, but on-scene time was comparable with that in the urban areas of Seiwa and Chuwa. The longest on-scene time was observed in Tohwa area.

**Table 3:** Differences among medical administrative regions

	Seiwa		Chuwa		Tohwa		Nanwa		*P* value
	*n*=4208		*n*=8435		*n*=4793		*n*=1705		
Age, years	48	(25-67)	46	(24–67)	46	(23–66)	51*	(27–68)	*P*<0.001
Male patients, n (%)	2372	(56.4)	4533	(53.7)	2741	(57.2)	642	(37.7)	*P*<0.001^¶^
High-risk injury, n (%)	208	(4.9)	377	(4.5)	322	(6.7)	184	(10.8)	*P*<0.001^¶^
Response time, min	9	(7–11)	8	(7–10)	9	(7–11)	10*	(7–15)	*P*<0.001
On-scene time, min	19	(14–25)	20	(15–26)	22*	(17–29)	20	(15–27)	*P*<0.001
Transport time, min	8	(5–12)	9	(6–14)	13	(8–19)	18*	(12–36)	*P*<0.001
EMS phone calls until hospital acceptance	1*	(1–1)	1	(1–2)	1	(1–2)	1	(1–2)	*P*<0.001

¶Chi-square test

*When compared to other areas by Kruskal–Wallis test with Bonferroni adjustment. EMS, emergency medical service

### GLMM for on-scene time

The results of the influencing factors of on-scene time are shown in Table 4. GLMM analysis for variables associated with on-scene time showed that calls for hospital acceptance accounted for 5.69 minutes per call. High-risk injury also accounted for 2.78 additional minutes to on-scene time. Age ≥65 years was associated with an additional 1.51 minutes to on-scene time. When the accident occurred after midnight and on a holiday, it required an additional 1.19 and 0.42 minutes to on-scene time, respectively. Vital sign variables were not significantly associated with on-scene time, except for the level of consciousness. Specifically, eye opening response to pain stimuli or no response lessened on-scene time by 1.99 minutes. Random effects showed that there was an up to 2-minute regional difference.

**Table 4:** Influencing factors of on-scene time in the generalized linear mixed model analysis

Variables			Estimate	95% CI	*P*
Intercept			16.5	14.7 to 18.4	*P*<0.001
**Fixed effects**					
	Elderly age		1.51	1.23 to 1.78	*P*<0.001
	Sex				
		male	0.18	-0.07 to 0.42	*P*=0.151
		female	Ref		
	High-risk injury		2.78	2.19 to 3.38	*P*<0.001
	Number of EMS phone calls until hospital acceptance		5.69	5.50 to 5.88	*P*<0.001
	Time Category				
		08:00-23:00	ref		
		00:00-07:00	1.19	0.80 to 1.60	*P*<0.001
	Holiday		0.42	0.16 to 0.68	*P*=0.002
	Consciousness level of eye opening by pain stimuli or no response		-1.99	-3.46 to -0.95	*P*=0.014
	SBP <90 mmHg		-0.14	-0.77 to 0.49	*P*=0.666
	HR <50 or ≥ 120 beats/min		0.25	-0.29 to 0.79	*P*=0.357
	RR <10 or ≥ 30 breaths/min		-0.4	-0.94 to 0.14	*P*=0.142
**Random effects**					
	Area				
		Seiwa	-1.01	-2.19 to 0.17	*P*=0.094
		Chuwa	-0.94	-2.12 to 0.23	*P*=0.114
		Tohwa	1.36	0.18 to 2.54	*P*=0.024
		Nanwa	0.6	-0.61 to 1.80	*P*=0.330
AIC		136076.109			
BIC		136091.803			

*SBP* systolic blood pressure, *DBP* diastolic blood pressure, *HR* heart rate, *RR* respiratory rate

## Discussion

The factors influencing EMS on-scene time in RTIs have not been clarified. The current study found that two or more EMS phone calls until hospital acceptance, high-risk injury, age ≥65 years, nighttime occurrence of RTI, and level of consciousness (specifically, eye opening response to pain stimuli or no response) are the major influencing factors of EMS on-scene time in RTIs. There were also differences in the on-scene time among the four regions evaluated, with differences of up to 2 minutes. Given that on-scene time is one of the indicators for EMS activities, these data can help EMS agencies develop policies for improving the outcomes of RTIs.

The strongest factor affecting on-scene time in this study was the number of EMS phone calls until hospital acceptance, which is consistent with the results of previous reports [11, 19]. Nagata et al examined all internal and external causes of critical emergencies in Kawasaki, Japan, and found that the number of EMS phone calls had odds ratio of 2.57 per call for on-scene time over 30 minutes [19]. Several factors can influence multiple phone calls for hospital acceptance. Katayama et al. examined factors associated with four or more EMS phone calls for traffic accident trauma patients and noted that nighttime hours and holidays were associated with multiple EMS phone calls [11]. These factors were also relevant in the present study, although they had minimal impact on on-scene time. Even for high-risk injury, 26.9% of cases required two or more calls until hospital acceptance. Difficulty in finding a hospital to transport to has a critical impact on the outcome following RTI. Similar to hospital diversion in western countries [20], this situation has a significant impact on time to definitive treatment for RTI patients. This issue should be solved within EMS systems to ensure immediate transport for RTI victims.

The current study also found that even high-risk injuries, which in many cases can result in critical injuries, require an additional 2.78 minutes (95% CI: 2.19 to 3.38) to on-scene time. Although the detailed analysis for this time factor was not available due to limited data, it is assumed that extrication of the patients, patient assessment, and protocol-based procedures might have accounted for this time. Previous reports from western countries indicate that interventions such as chest tube drainage or surgical airway can significantly prolong the on-scene time [12, 21]. In Japan, it is unlikely that these procedures account for the on-scene time because ELTs are not legally allowed to perform these procedures except for a physician-directed IV insertion for patients with hypotension.

Older age is also known to be associated with longer on-scene time [3, 22]. Age-related cognitive bias is one reason; another is failure of triage criteria to correctly identify elderly patients as they may sometimes appear to have less severe injury [23], even though this population can be severely injured from a less traumatic mechanism of injury [24]. Meanwhile, we found no association between vital signs on the acuity scale and on-scene time, consistent with previous results [3, 6]. On the other hand, the level of consciousness evaluated by eye opening response with pain stimuli or no response was strongly associated with on-scene time. Patients with this level of consciousness were more likely to be transported 2 minutes earlier. Previous studies report that a consciousness level of 2 or 3 in the JCS is associated with poor neurological outcomes [17, 18], and the Japanese EMS system generally accepts this level of consciousness as a sign of critical injury and the need for immediate transport. Another notable finding in our study is the difference in on-scene time among the four study regions, even between the rural regions. Studies that compared on-scene time in urban and rural areas found that the time interval is generally longer in rural areas [10, 25]. A study by Ashburn et. al., which examined EMS records of trauma patients in five counties in North Carolina, found regional differences of up to 6 minutes in EMS on-scene time [26]. However, these regional differences are not well explored, although various complicating factors could account for these differences. Levita et. al. interviewed paramedics on factors affecting on-scene time and found that scene characteristics (patient, bystander, location or weather), allied service (police or fire crews), accuracy of information from dispatch, ability to manage the scene, equipment, and implementation of policies are the barriers to reducing on-scene time [27]. In the current study, on-scene time was the longest in the Tohwa area. One distinct feature of this area in comparison to the other areas is that a long highway runs through mountains; however, the influence of this factor is difficult to quantify and examine. Since we controlled for these factors as a random effect in the analysis, our finding encompasses these factors with respect to the regional differences in on-scene time.

This study has inherent limitations to consider. First, the effect of unmeasured confounding factors could not be ruled out owing to the retrospective nature of the study. Second, the study data source did not contain information on injury severity and patient outcomes; thus, we were unable to investigate the association of on-scene time with other outcome measures. Therefore, our findings should be interpreted with caution. Finally, although there were differences in on-scene time among the study regions, the causes of these differences were not evaluated.

Future detailed studies on on-scene time regarding regional differences will help determine the critical factors that contribute to these variations and their impact on the emergency medical care system.

Despite the above limitations, we have identified the factors that can be associated with longer on-scene time. Our study findings can help EMS agencies develop future policies for immediate transport of RTI victims in the communities.

## Conclusions

The number of EMS phone calls until hospital acceptance is the most significant factor in reducing on-scene EMS time among RTI victims. High-risk injury or age ≥65 years are other significant factors. In contrast, JCS conscious level of 2-3 reduces on-scene time. Developing future EMS strategies based on these findings can improve the outcomes of RTI patients.

## Supplementary Information

**Additional file 1.** 

## Data Availability

The study dataset generated and analyzed during the current study is available from the corresponding author upon reasonable request.

## Abbreviations

ELT: emergency life-saving technician
e-MATCH: emergency Medical Alliance for Total Coordination of Healthcare
EMS: emergency medical service
EMT: emergency medical team
GLMM: Generalized linear mixed model
JCS: Japan Coma Scale
RTI: road traffic injury
