# Supplementary material for: Factors associated with EMS on-scene time and its regional difference in road traffic injuries: a population-based observational study
Source: BMC Emerg Med. 2022 Sep 15;22:160. doi: 10.1186/s12873-022-00718-1 (PMC9479253; doi:10.1186/s12873-022-00718-1)
Supplement: Supplementary file 1 — Additional file 1. [file 12873_2022_718_MOESM1_ESM.docx]

**High-energy trauma definition**

Fatality in same vehicle

Passenger ejection from vehicle

Severe vehicle deformity

Vehicle roll over

Run over pedestrian or bicycle driver

Vehicle or motorcycle accident $\geq$30 km/h

Pedestrian or bicycle driver is away from vehicle $\geq$5 m

Driver is away from motorcycle

Victim’s torso is caught in a vehicle or other object
